# Prognosis of Laboratory-Confirmed Influenza and Respiratory Syncytial Virus in Acute Heart Failure

**DOI:** 10.3390/jcm10194546

**Published:** 2021-09-30

**Authors:** David Carballo, Nicolas Garin, Jérôme Stirnemann, Aline Mamin, Virginie Prendki, Philippe Meyer, Christophe Marti, Francois Mach, Jean-Luc Reny, Jacques Serratrice, Laurent Kaiser, Sebastian Carballo

**Affiliations:** 1 Service of Cardiology, Department of Medicine, Geneva University Hospitals, 1211 Geneva, Switzerland; david.carballo@hcuge.ch (D.C.); philippe.meyer@hcuge.ch (P.M.); francois.mach@hcuge.ch (F.M.); 2 Service of General Internal Medicine, Department of Medicine, Geneva University Hospitals, 1211 Geneva, Switzerland; nicolas.garin@hcuge.ch (N.G.); jerome.stirnemann@hcuge.ch (J.S.); christophe.marti@hcuge.ch (C.M.); jean-luc.reny@hcuge.ch (J.-L.R.); jacques.serratrice@hcuge.ch (J.S.); 3 Service of Infectious Diseases, Department of Medicine, Geneva University Hospitals, 1211 Geneva, Switzerland; aline.mamin@hcuge.ch (A.M.); virginie.prendki@hcuge.ch (V.P.); laurent.kaiser@hcuge.ch (L.K.)

**Keywords:** acute heart failure, influenza, RSV

## Abstract

Concomitant respiratory viral infections may influence clinical outcomes of acute decompensated heart failure (ADHF) but this association is based on indirect observation. The aim of this study was to evaluate the prevalence and impact of laboratory-confirmed influenza or respiratory syncytial virus (RSV) infection on outcomes in patients hospitalised for ADHF. Prospective cohort of patients hospitalised for ADHF with systematic influenza and RSV screening using real-time PCR on nasopharyngeal swabs. The primary outcome was all-cause mortality or readmission at 90 days. Among 803 patients with ADHF, 196 (24.5%) patients had concomitant flu-like symptoms of influenza. PCR was positive in 45 patients (27 for influenza, 19 for RSV). At 90 days, PCR positive patients had lower rates of all-cause mortality or readmission as compared to patients without flu-like symptoms (HR 0.40, 95% CI 0.18–0.91, *p* = 0.03), and non-significantly less all-cause mortality (HR 0.30, 95% CI 0.04–2.20, *p* = 0.24), or HF-related death or readmission (HR 0.36, 95% CI 0.13–0.99, *p* = 0.05). The prevalence of influenza or RSV infection in patients admitted for ADHF was low and associated with less all-cause mortality and readmission. Concomitant viral infection with ADHF may not in itself be a predictor of poor outcomes. (ClinicalTrials.gov NCT02444416).

## 1. Introduction

Acute decompensated heart failure (ADHF) is a leading cause of hospitalization and mortality in patients older than 65 years and prognosis remains poor [1,2,3]. Influenza epidemics impact both general population mortality rates and number of hospitalizations [4,5]. Prognosis of heart failure (HF) is associated with factors such as age, renal function, blood pressure, left ventricular ejection fraction (LVEF), B-type natriuretic peptide (BNP) levels and certain common comorbidities [6,7,8,9,10]. Influenza has been considered another factor that could impact the clinical outcome of HF. However both the prevalence of influenza and its impact on outcomes have been insufficiently studied in patients with ADHF [11,12,13,14]. In a retrospective analysis of a national registry of patients hospitalised with influenza, 20% had concomitant HF but diagnosis of influenza was only based on International Classification of Diseases codes [12]. Furthermore, it has been suggested that patients with HF have a greater risk of hospitalization during the influenza season, the latter also characterized by an increase in the incidence of infections linked to other respiratory viruses [13,14,15]. Respiratory viruses such as respiratory syncytial virus (RSV), may affect cardiovascular outcomes in ADHF but have also been poorly evaluated [16]. Whilst influenza-like illness is a recognized syndrome, the association of laboratory confirmed influenza or RSV infection using polymerase chain reaction (PCR) with outcomes of patients hospitalised for ADHF has not previously been addressed [17].Influenza and RSV are the most common viral infections during seasonal epidemics and often have similar clinical presentations. Furthermore, these infections are seldomly microbiologically confirmed. The purpose of this study was therefore to evaluate both the prevalence and the impact of laboratory confirmed influenza and RSV disease on clinical outcomes in patients with ADHF.

## 2. Materials and Methods

### 2.1. Patients

Patients admitted for ADHF to the Department of medicine at the University Hospitals of Geneva between November 2014 and May 2019 were included. Patients were defined as having ADHF if they were hospitalised principally with symptoms and signs of HF, elevated natriuretic peptides (b-type natriuretic peptide [BNP] levels > 100 ng/L, or N-terminal-pro-BNP [NT-pro-BNP] levels > 300 ng/L), and structural and/or functional abnormality on echocardiography based on the European Society of Cardiology (ESC) criteria [7,18]. For patients with a left ventricular ejection fraction (LVEF) < 40%, no additional echocardiographic criterion was used. For patients with a LVEF ≥40%, at least one of the following additional echocardiographic criteria had to be met: left atrial volume index (LAVI) > 34 mL/m^2^, left ventricular mass index (LVMI) ≥ 115 g/m^2^ for males and ≥95 g/m^2^ for females, or diastolic dysfunction defined as an E/e′ ≥13 and a mean e’ septal and lateral wall <9 cm/s. The protocol was approved by the institutional ethics committee (protocol CER 14-019, ClinicalTrials.gov NCT02444416), and all patients gave written informed consent.

The University Hospital of Geneva is the main surveillance center for seasonal viral infection and harbors the national reference laboratory. Viral screening is conducted for both epidemiological and communicable disease prevention policies. Year round testing is carried out with peak systematic viral screening on all patients with flu-like symptoms during the influenza season, as defined by the Swiss Federal Office of Public Health (FOPH). Symptoms prompting screening include fever, rhinitis, cough and myalgia, new onset of one or more respiratory symptoms or worsening of a chronic condition involving respiratory symptoms. Screening is carried out with PCR on nasopharyngeal or oropharyngeal swabs (NPS). The FOPH publishes weekly and annual reports of cases of influenza based on a sentinel surveillance system that documents the number of consultations for flu like symptoms [19]. The epidemic threshold is defined as 69 cases per 100,000 populations [19]. Both influenza and RSV having similar clinical presentations and we therefore analysed the impact of these combined documented viral infections.

In this study, collected data included age, gender, weight, smoking status, a full medical history and medical therapy at admission, throughout hospital stay and at discharge. Clinical presentation was also recorded at admission and throughout hospital stay and including New York Heart Association (NYHA) dyspnea class, and clinical parameters, such as blood pressure, heart rate, and weight. Customary investigations were carried out on all patients, including full blood count, urea and electrolytes, electrocardiography, and echocardiography. Echocardiography was analyzed by experienced staff cardiologists. Patients were classified according to ESC guidelines as having heart failure with either reduced ejection fraction (LVEF) (HFrEF) if left ventricular ejection fraction (LVEF) was <40%, mid-range ejection fraction (HFmrEF) if LVEF was between 40% and 49%, or preserved ejection fraction (HFpEF) if LVEF was ≥50% [7].

Respiratory viral infections are punctual events and often have short term implications, and therefore the impact on clinical outcomes of concomitant viral infection was assessed at 90 days. Patients were prospectively followed via their contact with treating physicians and the examination of hospital medical records. Follow-up data included mortality, readmission, clinical state, and medication.

The primary outcome was a composite of all-cause mortality or readmission at 90 days. Secondary outcomes were all cause mortality, as well as heart failure related mortality and readmission at 90 days. Death or readmission were considered as being due to heart failure, if the primary cause was linked to heart failure. Patients were categorized either symptomatic, with flu like symptoms, or without suggestive symptoms. Patients with flu-like symptoms during the influenza season were considered as being PCR positive or PCR negative based on results of PCR for Influenza A, Influenza B or RSV.

### 2.2. Nucleic Acid Analysis Using Multiplex Real-Time PCR

The NPS were performed on symptomatic individuals during the initial hospitalization. These were placed in COPAN^®^ 305 Universal Transport Medium (COPAN Italia spa, Brescia, Italy) and processed directly in the central virology laboratory. Nucleic acids were extracted with Qiasymphony (Qiagen, Hombrechtikon, Switzerland) using a Virus/Pathogen kit (937055, Qiagen). Real-time polymerase chain reaction (RT-PCR) was performed on a Viia7 thermocycler (Life Technologies, Carlsbad, California, USA). The FTD Respiratory pathogens panel (FTD-2, FastTrack Diagnostics, Esch-sur-Alzette, Switzerland) was used to target viruses including influenza A, A (H1N1) pdm2009 and B viruses and respiratory syncytial virus.

### 2.3. Statistical Analysis

We used mean for continuous data, and number (%) for categorical data, as appropriate. ANOVA tests were used for comparing quantitative data, and Chi-square or Fischer test for categorical comparisons. Survival analysis with Log-Rank test was used for the unadjusted analysis, Kaplan-Meier plots, and Cox proportional hazard models for multivariate exploration. The multivariate model included variables that were associated with outcome on unadjusted analysis (*p* < 0.2), as well as recognized variables identified in previous studies such as age, gender, diabetes, chronic obstructive pulmonary disease (COPD), kidney disease, anaemia, hypertension [20]. Statistical analysis was performed using the SPSS statistical software package, (version 25.1).

## 3. Results

Of 803 patients hospitalised with ADHF, 196 presented flu-like symptoms of influenza and underwent NPS. PCR was positive in 45 patients of which 27 were positive for influenza influenza (20 Influenza A and 7 Influenza B) and 19 were positive for RSV; 1 patient was positive for both Influenza and RSV. Mean age in the cohort was 77 years and was similar in patients with and without flu-like symptoms (Table 1). Baseline characteristics including gender, body mass index (BMI), LVEF were comparable across all groups. History of chronic obstructive pulmonary disease (COPD) was more present in the virus tested groups. With respect to clinical presentation, those who had a positive viral PCR had more severe dyspnea and higher temperature at admission, but other features such as blood pressure and heart rate were similar. C-reactive protein was higher in the tested groups, irrespective of PCR positivity. There was no significant differences in NT-proBNP, hemoglobin or leucocyte count between the groups. Heart failure medication was similar across the groups.

Of the 151 patients with flu-like symptoms who were PCR negative, concomitant respiratory or infectious diseases was documented in 79 of them (52.3%). There were 49 radiologically confirmed pneumonias, 12 acute exacerbations of COPD, 7 other acute respiratory conditions (1 pulmonary embolism, 1 exacerbation of bronchiectasis, 3 non-specific interstitial pneumonias (NSIP), 1 idiopathic pulmonary fibrosis, 1 interstitial pneumonia attributed to amiodarone toxicity), and 11 infections or inflammatory conditions.

### Survival Analysis

**Figure 1:**
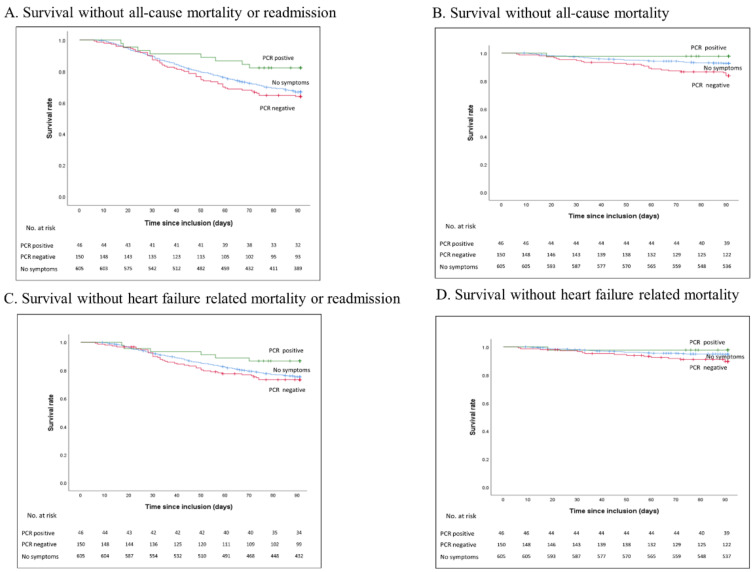
Kaplan-Meier survival analysis for 90-day clinical endpoints based on Influenza and respiratory syncytial virus (RSV) testing. Green: PCR positive, Red: PCR negative, Blue: patients without flu-like symptoms (no symptoms). (**A**). Survival without all-cause mortality or readmission, (**B**). Survival without all-cause mortality, (**C**). Survival without heart failure related mortality or readmission, (**D**). Survival without heart failure related mortality.

In survival analysis at 90 days, patients hospitalised for ADHF who were PCR positive for influenza or RSV, had fewer combined rates of all-cause mortality or readmission (HR 0.44, 95% CI 0.21–0.93, *p* = 0.03), and non-significantly less all-cause mortality (HR 0.31, 95% CI 0.42–2.23, *p* = 0.24), or HF related death or readmission (HR 0.44, 95% CI 0.18–1.07, *p* = 0.07) (Table 2 (unadjusted) and Table 3, Figure 1), as compared to patients without flu-like symptoms. PCR-negative patients had a trend toward higher all-cause mortality or readmission rates (HR 1.14, 95% CI 0.85–1.54, *p* = 0.38) and had higher all-cause mortality rates (HR 2.27, 95% CI 1.28–3.73, *p* = 0.001) as compared to patients without flu-like symptoms. There was also a trend for HF related death or readmission (HR 1.15, 95% CI 0.81–1.64, *p* = 0.07) and increased HF related death (HR 2.00 95% CI 1.08–3.71, *p* = 0.03) (Table 3, Figure 1). Overall, at 90 days there were 69 all-cause deaths, 262 all-cause deaths or readmissions, 47 heart failure related deaths, and 191 heart failure related deaths or readmissions. Survival analysis for the primary endpoint was similar when influenza and RSV were considered separately (HR 0.41, 95% CI 0.15–1.11, *p* = 0.08 and HR 0.44, 95% CI 0.14–1.37, *p* = 0.16 respectively). Variables that were included in the multivariate analysis included viral PCR status, as well as age, sex, BMI, hypertension COPD, diabetes, chronic kidney disease, chronic anaemia and LVEF (<40%) either because of significant association on univariate analysis, or because they are established predictors of outcome [8].

In multivariate adjusted analysis, PCR positive patients also had less all-cause mortality or readmission (HR 0.40, 95% CI 0.18–0.91, *p* = 0.03) as well as less HF related death or readmission as compared to patients without flu-like symptoms (HR 0.36, 95% CI 0.13–0.99, *p* = 0.05) (Table 3). As in univariate analysis, PCR negative patients, showed a trend towards higher all-cause mortality and readmission (HR 1.11, 95% CI 0.80–1.53, *p* < 0.66) (Table 2), as well as increased all-cause mortality (HR 2.41, 95% CI 1.40–4.15, *p* < 0.01) as compared to patients without flu-like symptoms.

A history of chronic obstructive pulmonary disease (COPD) was associated with increased all-cause death and readmission (HR 1.70, 95% CI 1.23–2.36, *p* < 0.01), as well increased heart failure related death or readmission (HR 1.84, 95% CI 1.27–2.67, *p* < 0.01).

Of the 27 patients with laboratory confirmed influenza infection, 17 received oseltamivir. Interaction variables were tested between oseltamivir use and virus testing categories and were non-significant. (*p* = 0.348 for death or readmission, *p* = 0.608 for death) but this study is underpowered to fully address this issue.

## 4. Discussion

In patients admitted for ADHF, the prevalence of concomitant PCR confirmed influenza or RSV infection was low, even in symptomatic patients during the influenza seasons. Furthermore, these cases were associated with less all-cause and HF-related mortality or hospital readmission at 90 days. Patients who were PCR negative for viral infection had comparatively higher rates of clinical outcomes. In this population, concomitant viral infection with ADHF was not in itself a predictor or marker of poor outcomes. Our study is the first to our knowledge to prospectively evaluate the impact of concomitant PCR proven influenza and RSV infection in patients hospitalised for ADHF.

The association between and viral infection and heart failure has been documented epidemiologically. Pathophysiologically, infections including those with influenza and RSV, increase metabolic demands. Patients with heart failure have decreased circulatory reserve and, as in our cohort and others, are often elderly with multiple comorbidities [21]. These patients may not be able to meet these increased metabolic needs when infected. Furthermore the severity of infection and the specific increased burden may explain variation in outcomes [11]. The burden of influenza and RSV has previously been estimated but determining the total burden is complex because the attributable mortality depends on death categories [22]. Underlying respiratory or circulatory deaths are severely impacted by these viral infections. However, as in other studies, the precise definition of circulatory deaths and or the means for viral infection determination are often not described. In our cohort, documented viral infection was not associated with worse outcome. Several hypotheses may explain this unanticipated association of PCR-influenza or RSV infection with fewer clinical outcomes in ADHF, even if the baseline prognostic variables do not seem to indicate that patients with PCR-confirmed influenza or RSV where at lesser risk. This group had a well-defined potential viral infectious trigger of decompensation in comparison with patients with no flu-like symptoms, in whom the triggers may have been more diverse and may have played a role in the poor outcomes. In our study, ADHF patients with flu-like symptoms and PCR negative for influenza and RSV had increased rates of clinical outcomes. Although confirming bacterial infection in respiratory disease such as pneumonia is notoriously difficult, we suggest that the worse outcome in patients who tested negatively may be due to more severe respiratory disease, such as bacterial pneumonia [23,24,25]. In our cohort, of the patients who tested negative, 53% had conditions such as documented pneumonia, COPD exacerbation or acute respiratory conditions.

Although there is evidence that influenza affects patients with HF, this is mostly based on indirect evidence from studies that use diagnostic codes and seasonal epidemiological data to show this association, but which do not strictly define criteria for either HF or influenza infection. Stronger association is demonstrated between influenza and other cardiovascular disease such as myocardial infarction [26]. A retrospective case control, propensity matched study based on an inpatient database showed that in all-cause hospitalizations, patients with HF and concomitant influenza infection had a higher rate of in-hospital mortality rate and respiratory complications [27]. In a similarly designed case control study of patients hospitalised with influenza, an association was shown between the comorbid presence of HF and increased in-hospital mortality [12]. In another retrospective cohort, the influenza-attributable risk of hospitalization among adults with congestive HF was increased during the influenza season [14]. The manner in which the influenza season is defined can also influence this estimation [13]. Further evidence for the association of influenza and poor outcomes in patients with HF derives from observations that influenza vaccination is associated with a reduced risk of both all-cause and cardiovascular death [11]. In our cohort, although some patients with proven influenza or RSV infection may have respiratory symptoms that may be mistaken for heart failure, we feel this to be a minimal source of misdiagnosis because all fulfilled the most recent HFrEF and HFpEF diagnostic criteria with a HFA–PEFF score ≥ 5, including high BNP levels [18]. Our results, based on definitive HF and PCR confirmed viral infection, provides a more robust evaluation of the association between these diseases.

With respect to RSV, few studies have addressed the impact of infection in patients with heart failure [16]. Indirect evidence suggests that concomitant RSV infection may be present in 13 to 20% of patients, but these studies of temporally related diagnoses lack clear definitions of heart failure and methods for RSV diagnosis [28,29]. In our study only 2.7% of patients with heart failure and respiratory symptoms had PCR confirmed RSV infection.

In our study, a history of COPD was associated with worse clinical outcomes. HF and COPD share common risk factors and several studies suggest a prevalence of concurrent HF and COPD of between 10% and 40% [30,31]. Concurrent COPD has previously been shown to independently predict mortality in patients with HFrEF as well as HFpEF [10,30,31,32].

Screening for viral infection in hospitalised patients has epidemiological value, however, in our cohort, proven influenza or RSV infection was not associated with a worse prognosis. When considering the clinical implications, despite routine screening for influenza, the prevalence of influenza infection in patients admitted with ADHF was lower than previously estimated. Furthermore, whether targeted antiviral therapy has an impact in these cases is not known. The retrospective report mentioned earlier, using linked data for nationwide registries, suggested that influenza vaccination is associated with a reduced risk of all-cause and cardiovascular death in patients with heart failure but precise characteristics of the heart failure status were not available in that report and confounding by indication maybe an issue [11]. Vaccination status is not readily available in clinical files and not routinely collected, and was therefore not available in our cohort.

One limitation of our study is the presence of residual confounding because of its observational nature, and our findings are therefore limited to association and not causality. Other limitations include the single centre nature of the cohort and the sample size. However, our hospital is the principle primary care structure for a large population basin and therefore manages most cases of decompensated HF. The proportion of patients with a LVEF < 50% was 44%, and with HFrEF defined as LVEF<40% was just over 31%. This is similar to figures of other cohorts of acute heart failure [33]. In our cohort, the proportion of patients with ischaemic heart disease was about 35%, also similar to published figures in other cohorts [34]. Although we have no information about the exact percentage of vaccinated patients in our cohort, seasonal influenza vaccination of the elderly population in Switzerland is estimated at approximately 40% [35]. How this level of vaccination impacts outcomes is unclear. Although survival analysis for the primary endpoint was similar when influenza and RSV were considered separately, the number of events was small. These results merit verification in a larger cohort. A strength however of our study was the systematic PCR screening of patients with flu-like symptoms, although molecular testing has performance limitations linked to sensitivity and specificity characteristics [36]. Further strengths are its prospective design and the use of robust clinical and biological inclusion criteria based on accepted guidelines and definitions for the identification of patients with HF [7,9]. The cohort corresponds to a real-life representation of patients hospitalised with ADHF, characterized by frequent comorbidities, which may allow its results to be generalizable to a larger population.

## 5. Conclusions

In patients admitted for ADHF, prevalence of concomitant influenza or RSV infection was low and associated with less all-cause mortality and readmission at 90 days. Concomitant viral infection with ADHF may not in itself be a predictor of poor outcomes. Studies in larger cohorts are needed to further clarify the precise nature of this relationship.

## Figures and Tables

**Table 1:** Baseline characteristics.

	Symptomatic Patients (N = 196)		Patients without Suggestive Symptoms N (%) (N = 607)	
	PCR positive N (%) (N = 45)	PCR negative N (%) (N = 151)		*p*
Age (mean (SD))	77.9 (±10.3)	77.1 (±10.2)	77.2 (±11.2)	0.93
Sex				0.49
Male	23 (51)	84 (56)	354 (58)	
Female	22 (48)	67 (44)	253 (42)	
BMI (Kg/m^2^)	27.8 (6.0)	27.7 (7.2)	26.5 (6.3)	0.09
Left ventricular ejection fraction				0.71
Preserved (>50%)	23 (55)	81 (57)	322 (54)	
Mid-range (40–49%)	8 (19)	18 (13)	77 (13)	
Reduced (<40%)	11 (26)	43 (30)	198 (33)	
Comorbidities				
Hypertension	34 (76)	121 (80)	482 (19)	0.03
Diabetes	17 (39)	44 (29)	197 (33)	0.43
Chronic kidney disease	19 (48)	58 (39)	205 (36)	0.29
COPD	9 (21)	35 (24)	70 (12)	<0.001
Atrial fibrillation	21 (47)	70 (47)	276 (47)	0.99
Anaemia	21 (47)	62 (41)	245 (40)	0.06
Etiology of heart failure				
Ischaemic	15 (33.3)	57 (37.7)	192 (31.6)	0.05
Hypertensive	11 (24.4)	40 (26.5)	123 (20.3)	0.03
Valvular	13 (28.9)	29 (19.2)	128 (21.1)	0.05
Arythmic	16 (34.8)	50 (33.3)	180 (29.7)	0.07
Active smoking	10 (22)	23 (15)	102 (17)	0.55
NYHA class				0.01
I	0	1 (1)	6 (1)	
II	3 (7)	9 (6)	35 (6)	
III	6 (13)	45 (31)	219 (39)	
IV	36 (80)	90 (62)	296 (53)	
De novo heart failure	9 (20)	43 (29)	204 (34)	0.21
Parameters (mean (SD))				
Systolic blood pressure	143 (±28)	137 (±25)	142 (±27)	0.12
Diastolic blood pressure	77 (±18)	77 (±16)	83 (±20)	0.003
Heart rate	92 (±21)	95 (±59)	90 (±25)	0.19
Temperature (°C)	37.3 (±0.9)	36.9 (±0.8)	36.8 (±0.6)	<0.001
Fever (>38 °C) N(%)	10 (22.2)	14 (9.3)	22 (3.6)	<0.001
BNP (*n* = 123)	1549 (±1520)	1264 (±1563)	1150 (±1021)	0.53
NT-proBNP (*n* = 678)	7752 (±12060)	8979 (±12302)	8187 (±11091)	0.76
Hb (g/L)	125 (±26)	123 (±23)	124 (±23)	0.75
eGFR (mL/min)	51 (±23)	53 (±22)	53 (±24)	0.77
CRP (*n* = 786)	48 (±64)	55 (±74)	24 (±43)	<0.001
Leucocytes	8.7 (±3.6)	12.6 (±26.7)	10.6 (±19.6)	0.43
Medication at admission				
ACEi	11 (24)	42 (28)	166 (27)	0.52
ARB	19 (42)	40 (27)	177 (29)	0.19
Loop diuretics	24 (51)	88 (58)	268(44)	0.07
Betablockers	24 (53)	80 (53)	342 (56)	0.52
MRA	5 (11)	20 (13)	79 (13)	0.99
Medication at discharge				
ACEi	16 (36)	63 (42)	231 (38)	0.96
ARB	9 (20)	29 (19)	138 (23)	0.86
Loop diuretics	35 (78)	114 (76)	445 (73)	0.19
Betablockers	30 (67)	97 (64)	417 (69)	0.82
MRA	6 (13)	21 (14)	110 (18)	0.34

BMI: body mass index, COPD: chronic obstructive pulmonary disease, NYHA: New York Heart Association, BNP: Brain natriuretic peptide, Hb: hemoglobin, eGFR: estimated glomerular filtration rate, ACEi: angiotensin converting enzyme inhibitors, ARB: angiotensin II receptor blockers, MRA: mineralocorticoid receptor antagonist, HF: heart failure, PCR: polymerase chain reaction.

**Table 2:** Unadjusted analysis of association with 90 day all-cause mortality and readmission.

	All-Cause Mortality or Readmission (HR (95% CI)	*p* Value
Sex (male)	1.10 (0.86–1.40)	0.46
Age, year	1.00 (0.99–1.01)	0.70
BMI, Kg/m^2^	0.99 (0.97–1.01)	0.42
Hypertension	1.14 (0.84–1.56)	0.40
COPD	1.72 (1.27–2.33)	<0.001
Diabetes	1.13 (0.87–1.46)	0.36
Chronic kidney disease	1.25 (0.97–1.61)	0.08
Chronic anemia	1.25 (0.98–1.60)	0.08
LVEF (<40%) ^a^	0.94 (0.73–1.22)	0.66

^a^ As compared to preserved LVEF. HR: Hazard ratio, LVEF: left ventricular ejection fraction, BMI: body mass index, COPD: chronic obstructive pulmonary disease.

**Table 3:** Association of viral testing status with 90 day clinical outcomes expressed as hazard ratios in symptomatic patients as compared to patients without flu-like symptoms.

	Symptomatic Patients			
	PCR Positive Patients HR * (95% CI) *p* Value		PCR Negative Patients HR * (95% CI) *p* Value	
	Unadjusted	Adjusted	Unadjusted	Adjusted
All-cause mortality or readmission	0.44 (0.21–0.93) 0.03	0.40 (0.18–0.91) 0.03	1.14 (0.85–1.54) 0.38	1.11 (0.80–1.53) 0.66
All-cause mortality	0.31 (0.42–2.23) 0.24	0.30 (0.04–2.20) 0.24	2.27 (1.28–3.73) 0.001	2.41 (1.40–4.15) <0.01
Heart failure related mortality or readmission	0.44 (0.18–1.07) 0.07	0.36 (0.13–0.99) 0.05	1.13 (0.70–1.60) 0.51	1.16 (0.80–1.68) 0.44
Heart failure related mortality	0.44 (0.06–3.19) 0.41	0.43 (0.06–3.20) 0.41	2.02 (1.09–3.74) 0.03	2.00 (1.01–3.96) 0.05

HR: Hazard ratio *as compared to patients without flu-like symptoms. The model was adjusted for age, sex, chronic obstructive pulmonary disease, chronic kidney disease, chronic anaemia, hypertension, diabetes and left ventricular ejection fraction.

## Data Availability

The data presented in this study are available on request from the corresponding author.
